# Sex-biased TGFβ signalling in pulmonary arterial hypertension

**DOI:** 10.1093/cvr/cvad129

**Published:** 2023-08-18

**Authors:** Marius Wits, Clarissa Becher, Frances de Man, Gonzalo Sanchez-Duffhues, Marie-José Goumans

**Affiliations:** Department of Cell and Chemical Biology, Leiden University Medical Center, Einthovenweg 20, 2333 ZC Leiden, The Netherlands; Department of Cell and Chemical Biology, Leiden University Medical Center, Einthovenweg 20, 2333 ZC Leiden, The Netherlands; Department of Pulmonary Medicine, Amsterdam University Medical Center (UMC) (Vrije Universiteit), 1081 HV Amsterdam, The Netherlands; Department of Cell and Chemical Biology, Leiden University Medical Center, Einthovenweg 20, 2333 ZC Leiden, The Netherlands; Nanomaterials and Nanotechnology Research Center (CINN-CSIC), Health Research Institute of Asturias (ISPA), 33011 Oviedo, Spain; Department of Cell and Chemical Biology, Leiden University Medical Center, Einthovenweg 20, 2333 ZC Leiden, The Netherlands

**Keywords:** Activin, Androgen, BMP, BMPR2, Endothelial, Oestrogen, HHT, Hypertension, PAH, TGFβ

## Abstract

Pulmonary arterial hypertension (PAH) is a rare cardiovascular disorder leading to pulmonary hypertension and, often fatal, right heart failure. Sex differences in PAH are evident, which primarily presents with a female predominance and increased male severity. Disturbed signalling of the transforming growth factor-β (TGFβ) family and gene mutations in the bone morphogenetic protein receptor 2 (*BMPR2*) are risk factors for PAH development, but how sex-specific cues affect the TGFβ family signalling in PAH remains poorly understood. In this review, we aim to explore the sex bias in PAH by examining sex differences in the TGFβ signalling family through mechanistical and translational evidence. Sex hormones including oestrogens, progestogens, and androgens, can determine the expression of receptors (including BMPR2), ligands, and soluble antagonists within the TGFβ family in a tissue-specific manner. Furthermore, sex-related genetic processes, i.e. Y-chromosome expression and X-chromosome inactivation, can influence the TGFβ signalling family at multiple levels. Given the clinical and mechanistical similarities, we expect that the conclusions arising from this review may apply also to hereditary haemorrhagic telangiectasia (HHT), a rare vascular disorder affecting the TGFβ signalling family pathway. In summary, we anticipate that investigating the TGFβ signalling family in a sex-specific manner will contribute to further understand the underlying processes leading to PAH and likely HHT.

## Graphical Abstract

**Graphical Abstract:**
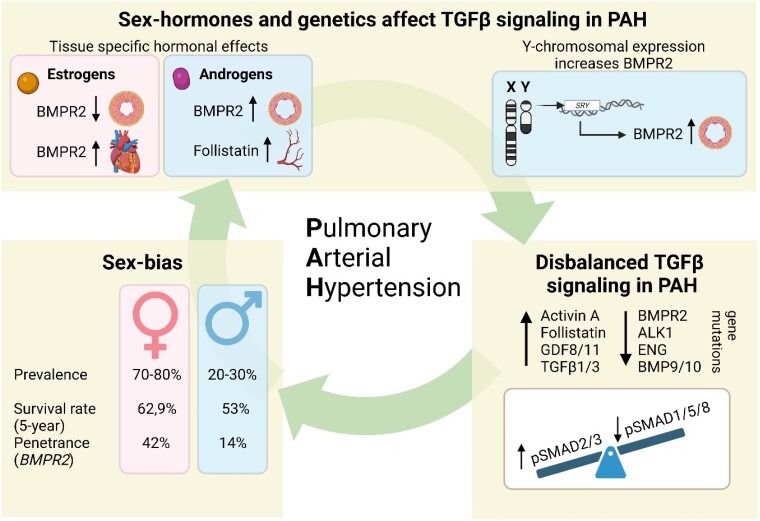

**Time of primary review: 20 days**

## 1. Introduction: pulmonary arterial hypertension

Pulmonary arterial hypertension (PAH) belongs to Group I in the total of five (I–V) groups of pulmonary hypertension. Group I is substratified in, among others, idiopathic PAH (IPAH) and heritable PAH (HPAH). HPAH has a known genetic origin, by either familial contribution or genetic correlation,^1^ while IPAH has an un-familial cause at the time of diagnosis. As established in the 2022 ESC/ERS Guidelines for the diagnosis and treatment of pulmonary hypertension, pre-capillary PH (including PAH) is defined by a mean pulmonary arterial pressure (mPAP) of >20 mmHg, pulmonary arterial wedge pressure (PAWP) of ≤15 mmHg, and pulmonary vascular resistance (PVR) of >2 Wood Units (WU).^2^ The increased workload on the right heart causes ventricular dilatation and hypertrophy, resulting in progressive right heart failure. Pulmonary vascular remodelling constitutes the main pathological event at the onset of PAH. Remodelling of the distal pulmonary arteries involves abnormal proliferation of endothelial cells (ECs), smooth muscle cells (SMCs), and fibroblasts; apoptosis resistance of ECs; excessive EC migration that becomes dysfunctional, in part due to endothelial-to-mesenchymal transition (EndMT) (distal); migration of SMCs (proximal); inflammatory influx of macrophages and lymphocytes; and the formation of plexiform lesions.^3–5^

Although PAH is a disease caused by remodelling of the pulmonary vasculature, end-stage patients ultimately die from right heart failure.^2^ To date, there is no approved treatment curing or reversing disease progression. The current treatment of PAH mainly consists of the single or combined administration of pulmonary vasodilators acting on the guanylate cyclase, endothelin, or prostacyclin pathways,^6^ only postponing further progression and eventually requiring lung transplantation in severe cases.^7^ Recently, the Phase 3 clinical trial STELLAR has concluded excellent clinical outcomes in PAH patients using Sotatercept.^8^

Sex-related differences in disease prevalence and severity are known for PAH. The US REVEAL study showed that 80% of the PAH patients are women (4:1 ratio).^9,10^ Comparably, multiple registries across Europe concluded a female bias in PAH of approximately 70% (2.3:1 ratio).^11–16^ Interestingly, the disease bias towards women declines by age when comparing age groups 18–65 with >65 years old in IPAH patients.^12^ In addition, PAH disease penetrance is also defined by sex, with a 42% in females over 14% in male HPAH patients.^17^ Remarkably, diagnosed PAH male patients are more severely burdened, with nearly a 10% reduction in 5-year survival rate (53%) compared to females (62.9%).^9^

The underlying cellular and molecular causes of these sex-related differences in PAH have not yet been fully understood, although many hypotheses have been proposed. These often involve hormonal-based alterations, although metabolism, genetics, and/or the immune system might also play a role.^18–20^ In general, androgens are considered vasculo-protective and a contributor to pulmonary vasodilation,^21^ perhaps underlying the female predominance in PAH. On the other side, oestrogens have been reported to be vasculo-protective in coronary heart disease in women (reviewed in reference ^22^). In PAH, oestrogens promote right ventricle adaptation in women,^23^ which might lead to a less severe phenotype compared to men.^24^ Further, chromosomal differences also play a role, for instance, the Y-chromosome is thought to have vascular protective gene expression profiles in PAH.^25^ In this review, we further discuss if sex determinants, i.e. sex hormones and -chromosomal effects, are a driver of PAH development by altering transforming growth factor-β (TGFβ) signalling.

## 2. Transforming growth factor-β signal transduction

Disturbances in the TGFβ signalling family contribute to PAH disease development and progression.^26–28^ The TGFβ family pathway drives developmental processes and tissue homeostasis^29^ within the cardiovascular system.^28,30^ In mammals, the TGFβ family is comprised of 33 structurally related polypeptides, including the TGFβ1–3 isoforms, the bone morphogenetic proteins (BMP1–15), nodal, the growth and differentiation factors (GDFs), the activins and inhibins, and the anti-Müllerian hormone (AMH).^31–37^ The TGFβ ligands exert pleiotropic effects by controlling cell proliferation, migration, and differentiation in a spatial and temporal manner.^29^ Disturbed signalling can result in cancer,^38^ musculoskeletal disorders,^39^ fibrosis,^40^ and cardiovascular diseases^28,41–43^.

Most TGFβ family members, with BMPs being the exception,^44^ are secreted in an inactive form within a latent complex (reviewed in reference ^45^). These large latent complexes include the mature TGFβ polypeptide shielded by latency-associated peptides and latent TGFβ binding proteins.^46^ These additional factors also bind to the extracellular matrix (ECM) or the plasma membrane via receptors like glycoprotein-A repetitions predominant (GARP), creating an ECM storage of accumulated latent TGFβ. The mature TGFβ polypeptides are released via several mechanisms allowing a quick functional response on demand.^45^

**Figure 1:**
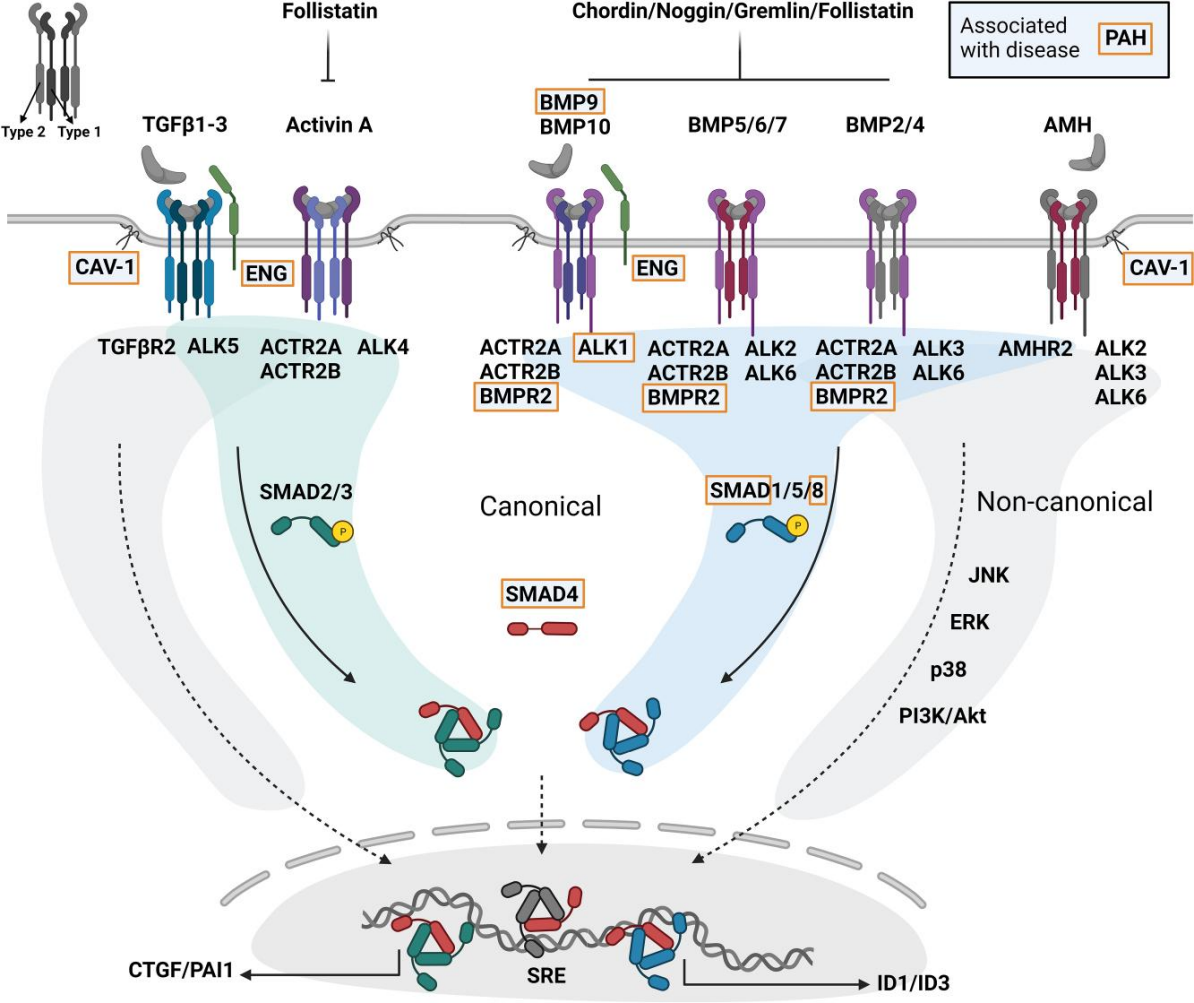
Schematic representation of the TGFβ signalling family. Ligands of the TGFβ family (TGFβ1–3, Activin A, BMP2/4/5/6/7/9/10, AMH) bind their type I (ALK1/2/3/4/5/6) and II (TGFβR2, ACTR2A/B, BMPR2, AMHR2) plasma membrane receptors. Soluble antagonists (Follistatin, Chordin, Noggin, Gremlin) can decrease ligand accessibility. Type III receptors (i.e., endoglin) can further regulate ligand–receptor complex formation. Upon Type I receptor activation, the intracellular signalling molecules (R-SMADs) are phosphorylated and form a heterotrimeric complex with SMAD4. ALK4/5 (stimulated by TGFβ/Activin A ligands) signal via SMAD2/3 whereas ALK1/2/3/6 (stimulated by BMP/AMH ligands) signal via SMAD1/5/8. R-SMAD/SMAD4 complexes translocate to the nucleus to regulate the activity of gene promoters. Also non-canonical signalling (JNK, ERK, p38, PI3K/Akt) can occur via TGFβ signalling. Mutations in genes encoding TGFβ factors have been linked to PAH development. Not all factors within the TGFβ signalling family have been incorporated in the figure for clarity purposes. PAH, pulmonary arterial hypertension; TGFβ, transforming growth factor-β; BMP, bone morphogenetic protein; AMH, anti-Müllerian hormone; CAV-1, caveolin-1; ENG, endoglin; ALK, activin receptor-like kinase; TGFβR2, TGFβ receptor 2; ACTR2, activin receptor Type II; BMPR2, BMP receptor Type II; SMAD, small mothers against decapentaplegic; JNK, c-jun N-terminal kinase; ERK, extracellular signal-regulated kinase; PI3K, phosphoinositide 3-kinase; SRE, SMAD responsive element.

**Figure 2:**
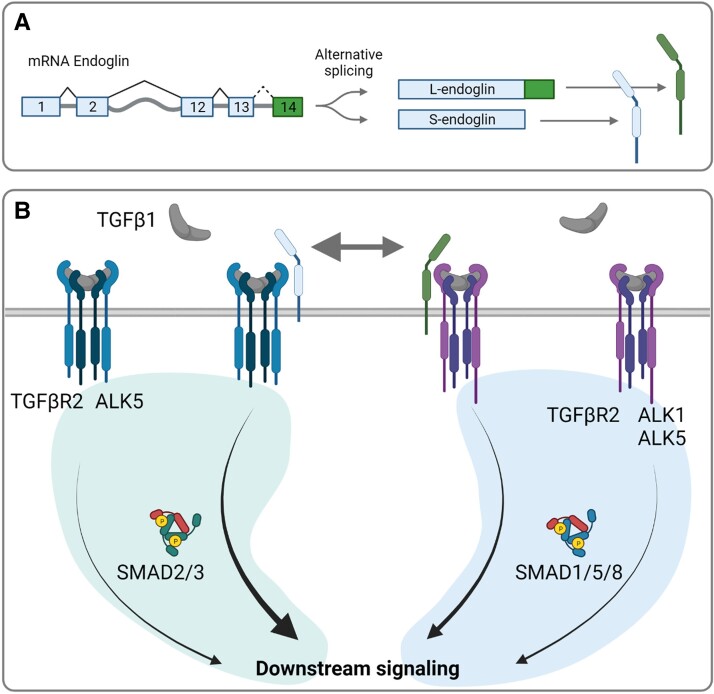
A schematic depiction of the splice variants (*A*) and signalling function (*B*) of endoglin on TGFβ1 signalling. The short (S-) and long (L-)endoglin variants are alternatively spliced by excluding or including exon 14, respectively (*A*). Both S- and L-endoglin increases TGFβ1 signalling; however, S-endoglin favours ALK5 signalling where L-endoglin favours ALK1 dependent signalling (*B*). Therefore, as observed by,^55,56^ a balance shift towards S-endoglin increases TGFβ signalling by SMAD2/3 phosphorylation. TGF, transforming growth factor; ALK, activin-like kinase; SMAD, small mothers against decapentaplegic.

Active TGFβ ligands signal via a heterotetrameric complex of Type I and II serine–threonine kinase receptors (*Figure 1*).^47^ In vertebrates, seven activin like kinase (ALK)1-7 Type I receptors and five Type II receptors (TGFβ receptor 2 (TGFβR2), activin receptor 2A (ACVR2A), ACVR2B, bone morphogenetic protein receptor 2 (BMPR2), and anti-Müllerian hormone receptor 2 (AMHR2)) exist. Since the ligands of the TGFβ family bind with different affinities to their receptor complexes, the relative expression level of the TGFβ family receptors may determine sensitivity of a particular cell type or tissue to a TGFβ ligand.^48^ Overall, TGFβs and activins bind with a high affinity to their Type II receptors, whereas BMPs and GDFs exhibit a high affinity for their Type I receptors.^49^ Co-receptors like TGFβR3 (betaglycan) or endoglin (*Figures 1* and *2*) can enhance ligand binding to Type I/II receptors when membrane bound, but can act as ligand trap when secreted in a soluble form.^50^ Next to these accessory proteins, soluble signalling modulators including Noggin, Gremlin, and Follistatin also exert regulatory effects on the TGFβ family signalling as ligand agonists or antagonists.^51^

Upon ligand–receptor interaction and receptor complex formation, the constitutively active Type II receptor phosphorylates and activates the Type I receptor. Next, the Type I receptor kinase initiates the signal transduction cascade by phosphorylating intracellular downstream proteins, i.e. receptor regulated-SMADs (R-SMADs) (*Figure 1*). Generally, TGFβ1–3 and Activins signal by SMAD2/3 phosphorylation whereas BMPs, GDFs, and AMH signal via phosphorylation of SMAD1/5/8. In the vasculature for instance, BMP9 and -10 are important factors necessary for endothelial homeostasis, exhibiting a high affinity for BMPR2/ALK1 receptor complexes, mainly expressed in ECs.^52,53^ Both ALK1/SMAD1/5/8 and ALK5/SMAD2/3 signalling are co-regulated by endoglin in ECs.^54^ Interestingly, the two splice variants short- and long-endoglin favour different Type I receptors, being S-endoglin pro-ALK5 and L-endoglin pro-ALK1 (*Figure 2*).^55^

Once phosphorylated, the R-SMADs bind to the co-SMAD SMAD4 and form heterotrimeric complexes. Furthermore, Inhibitory SMADs (I-SMADs, SMAD6 and 7) are transcriptional targets of the TGFβ superfamily and create a classical negative feedback loop interacting with and promoting the degradation of TGFβ receptors by e.g. SMURF1/2.^57,58^

SMAD4-containing heterotrimeric complexes translocate to the nucleus, where they associate with cell type- and pathway-induced transcription factors to modulate target gene expression.^59^ Different DNA motifs on the regulatory regions of genes have been described for the SMAD4, SMAD2/3, and SMAD1/5/8.^57,60–62^ The binding affinity of SMADs for DNA is relatively low and can be enhanced through association with other transcription factors, which may determine cell-type-specific TGFβ responses.^57^ Therefore, the transcriptional activity induced by ligands of the TGFβ superfamily can be ‘fine-tuned’ at multiple levels, including the relative expression levels of ligands, (co)receptors, (ant)agonists, and nuclear transcription factors that are activated in a tissue and stimulus-dependent manner.^57,63^ Many of the cell-type-specific responses to TGFβ ligands are attributed to the so-called non-canonical pathways. The non-canonical signalling may not require the Type I receptor kinase activity.^64^ Furthermore, although the TGFβ Type I and II receptors are known serine/threonine kinases, they can also phosphorylate tyrosine residues and act as dual-specificity kinases. Therefore, tyrosine phosphorylation may be an alternative route to mediate SMAD-independent signalling.^65^ TGFβ non-canonical signalling is often highly context dependent. For example in vascular settings, TGFβ-induced EndMT is also mediated through the activation of extracellular signal-regulated kinase (ERK)^66^ and c-Jun *N*-terminal kinase (JNK).^67^ Further, TGFβ-mediated inhibition of primary vascular smooth muscle cell proliferation has been demonstrated to be p38-dependent.^68^ Unfortunately, much is still to be deciphered in the context of non-canonical TGFβ signalling and PAH. Accordingly, in this review, we mainly focus on canonical signalling of the TGFβ family.

## 3. The TGFβ signalling family in PAH

PAH is linked to disturbances within the TGFβ signalling family pathway. Mutations in genes encoding for components of the TGFβ signalling cascade have been identified, such as *ACVRL1* (encoding ALK1), *ENG* (encoding endoglin), *SMAD9* (encoding SMAD8),^69,70^*SMAD1*,^69^*SMAD4*,^69^ and *GDF2* (encoding BMP9)^71^ (*Figure 1*). The most relevant gene mutation by far involves the *BMPR2* gene, which is affected by loss of function mutations in 70–80% of the HPAH and in 10–20% of the IPAH patients.^72^ Additionally, mutations in genes not part of the canonical TGFβ signalling cascade have also been reported (i.e. *CAV1*,^73^*TBX4*,^74^*EIF2AK4*,^75^ and *KCKN3*^76^).

Currently, more than 650 different *BMPR2* mutations have been described.^77–79^ These mutations may occur in non-coding regions but are mostly located in the coding regions containing the extracellular, transmembrane, kinase, and cytoplasmic functional domains. Noteworthy, approximately 50% of total mutations are found in the kinase domain of BMPR2.^77,80^ The different gene mutations consist of single nucleotide substitutions, leading to non-sense, missense, or splice site mutations; and insertions or deletions causing small and partial insertions, deletions, or duplications. A study looking at 144 different *BMPR2* mutations from a broad international PAH patient cohort, predicted that around 70% of all the mutations result in non-mediated decay of the truncated transcripts.^80^ Follow-up studies concluded similar findings.^77^ The resulting haploinsufficiency is therefore the main cause of disrupted TGFβ signalling. Still, PAH penetrance is low in families with mutations causing haploinsufficiency. Comparing non-affected mutation carriers with PAH patients within the same family, Hamid *et al.*^81^ showed that the expression levels from the wild-type *BMPR2* allele impact disease progression, with lower BMPR2 expression levels observed in more affected individuals. Therefore, next to loss of BMPR2 due to genetic mutations, additional triggers to reduce endogenous BMPR2 expression are needed to result in pathogenic TGFβ signalling.

In HPAH patients carrying a *BMPR2* mutation, the BMPR2 and phosphorylated SMAD1/5/8 expression are decreased in lung tissues,^42,82,83^ consistent with a decreased expression of BMP transcriptional targets such as *ID3.*^84^ Interestingly, BMPR2 expression is also decreased in idiopathic patients,^82^ which might be due to (post)transcriptional inhibition of BMPR2 expression in inflammatory environments.^67,85^ Serum and lung expression of TGFβ1 and TGFβ3 ligands are increased in PAH patients,^86,87^ consistent with enhanced expression of a TGFβ target gene *SERPINE1.*^88^ Additionally, Activin A and its natural antagonist Follistatin and Follistatin Like-3 are both increased in serum of HPAH and IPAH patients,^89,90^ of which Activin A is known to be secreted by macrophages, bronchial epithelial cells, and lung microvascular ECs.^91^ Given the counterbalance between BMP and TGFβ signalling, it is well accepted that increased TGFβ and Activin A signalling in PAH results from inactivating mutations in BMP pathway components.^26,92^ However, recent publications have unveiled novel mechanisms triggered upon loss of BMPR2. Hiepen *et al.*^93^ recently showed that loss of BMPR2 in ECs results in the formation of a mixed-tetrameric receptor complex TGFβ-TGFβR2-ALK5 including a Type I BMP receptor. The inclusion of a Type I BMP receptor allows the activation of pSMAD1/5/8 signalling, while this is prevented by BMPR2 over-expression. Earlier work by other groups further strengthens this hypothesis of mixed-TGFβ/BMP receptor complexes and subsequent activation of pSMAD1/5/8 upon stimulation with TGFβ or Activins.^94–97^ This can be a very relevant mechanism in PAH, as not only TGFβ1, but also Activin A levels are increased in serum of IPAH and HPAH patients.^89,90^

Loss of function mutations in *ENG* have been found in familial PAH patients.^98^ IPAH patients display increased circulating and non-circulating endoglin levels,^86^ measured in serum and in isolated ECs, respectively. This increased soluble endoglin is related with disturbed EC function. Moreover, alternative splice variants of endoglin can shift the TGFβ/BMP signalling balance.^55^ These variants differ in exon 14, and result in L-endoglin and S-endoglin variants, where L-endoglin displays a longer intracellular domain.^99^ This intracellular domain contains phosphorylation sites for TGFβR2, ALK5, and ALK1.^100^ As shown by Lee *et al.*,^56^ increased short (S-)endoglin over long (L-)endoglin causes an increase in SMAD2/3 over SMAD1/5 phosphorylation in ECs (*Figure 2*). Interestingly, this disbalance may also occur in HPAH patients with mutations in exon 14 of the *ENG* gene, favouring the short splicing variant S-endoglin and therefore increasing TGFβ signalling.

Taken together, alterations in BMP receptor complexes due to, for example, loss of function mutations in *BMPR2* or *ENG*, can disbalance the cellular responses to the increased circulating levels of TGFβ/Activin ligands. Induction of BMP-driven pSMAD1/5/8 is often described as protective in PAH. However, pSMAD1/5/8 signalling resulting from TGFβ or Activins in the absence of BMPR2 may not be beneficial as well. One explanation might be that TGFβ and Activin may compete with canonical BMP ligands for the receptors, in this case inducing mixed-tetrameric receptor complexes. These mixed complexes may result in less potent or more transient pSMAD1/5/8 activation and different non-canonical signalling activation, compared with classical BMP-induced complexes. Further, it can lead to short-term signalling saturation (by e.g. SMAD4 competition). Therefore, comprehensive studies including not only BMPR2 downstream signalling but also other TGFβ branches in the context of PAH are needed, as all these different signalling branches may contribute to vascular remodelling and subsequent PAH development.^93^

**Figure 3:**
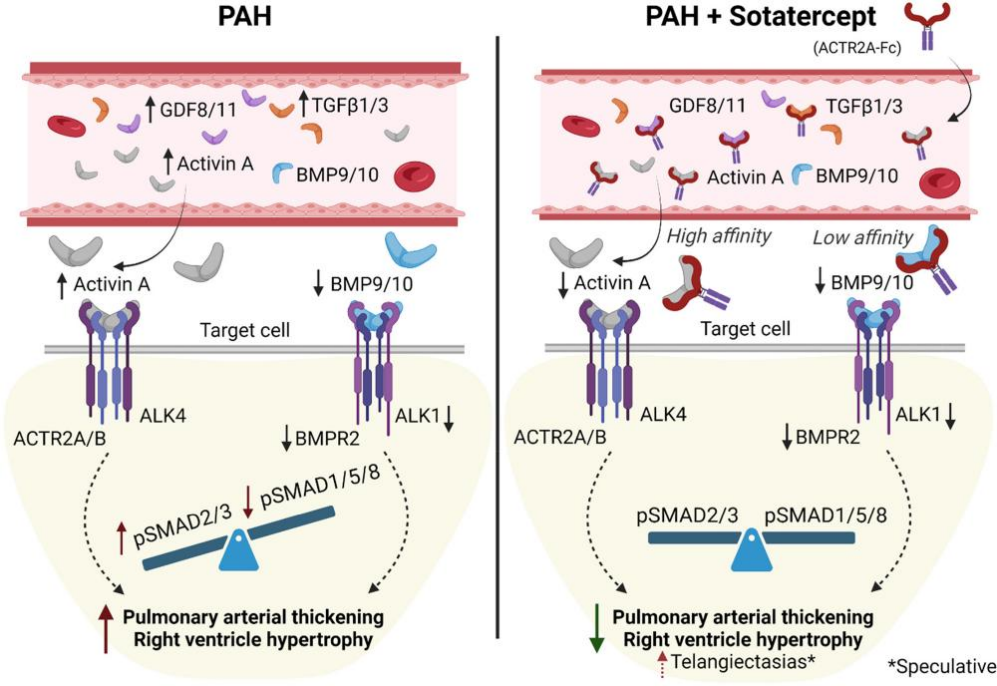
Sotatercept (ACTR2A-Fc) sequesters TGFβ ligands to restore the disbalanced signalling in PAH. The soluble ligands activin A, GDF8/11 and TGFβ1/3 are elevated in PAH causing increased SMAD2/3 phosphorylation over SMAD1/5/8 signalling. This disturbed TGFβ signalling underlies increased pulmonary arterial thickening with a subsequent rise in pulmonary arterial pressure and right ventricle hypertrophy. Treatment with Sotatercept normalizes the signalling imbalance by shielding soluble TGFβ ligands, resulting in a decrease in pulmonary arterial thickening and right ventricle hypertrophy. *Low affinity inhibition of BMP10 by Sotatercept might disturb endothelial homeostasis and subsequently causing telangiectasias. TGF, transforming growth factor; GDF, growth differentiation factor; BMP, bone morphogenetic protein; ALK, activin receptor-like kinase; ACTR2, activin receptor Type II; BMPR2, BMP receptor Type II; SMAD, small mothers against decapentaplegic.

In line with a prominent role of aberrant TGFβ signalling as underlying cause of PAH, the ACTR2A-Fc fusion molecule Sotatercept aims to counter this imbalance by trapping soluble TGFβ ligands (*Figure 3*) and thereby restoring pathogenic TGFβ signalling.^8,101^ Indeed, *in vitro* evidence shows that ACTR2A-Fc treatment of pulmonary ECs reduces pSMAD2/3 while enhances pSMAD1/5/8 signalling. Further, pulmonary arterial thickening and cardiac hypertrophy were partially restored by only 2–4 weeks of Sotatercept treatment in PH rat models.^101^ The Type II receptor ACTR2A is able to bind many different TGFβ ligands (*Figure 1*) with different affinities. High affinity ligands of ACTR2A include Activin A, GDF8, and GDF11,^49^ which levels are all increased in PAH.^89,90,101^ Due to the promiscuous role of ACTR2A in complex formation and binding capacity to many other ligands (also e.g. BMP10),^49^ we stress that Sotatercept’s success might rely on its unspecific targeting of TGFβ ligands. The balance of the combinatory levels of circulating TGFβ ligands in the patient and their differential affinities to Sotatercept therefore drives its pharmacological function. However, Sotatercept may also reduce BMP activity, which can underlie the undesirable side effects observed in PAH patients involved in a recent clinical trial (as reviewed in reference^102^). For instance, the inhibition of BMP10 by high doses of Sotatercept can interfere with BMP10 homeostatic function on the endothelium,^53^ maybe resulting in telangiectasias (*Figure 3*). Furthermore, thus far this drug has been tested in patients on background therapy. Whether a therapeutic approach based on solely targeting ACTR2A ligands is successful, remains to be investigated.

## 4. Sex hormones and the TGFβ signalling family

As aforementioned, disturbed signalling induced by TGFβ family members constitutes a hallmark in PAH. Given the sex bias observed in this disease, it becomes key to understand the mechanisms by which sex-specific cues may fine-tune the TGFβ family signalling. Sex hormones are derived from cholesterol. Female sex hormones are oestrogens and progestogens, including oestradiol and progesterone, respectively. Male hormones are androgens, of which testosterone is the dominant effector. Sex steroids induce signal transduction by binding to their soluble nuclear receptors; oestrogen receptor (ER), progesterone receptor (PR), and androgen receptor (AR). These receptors act as signal transducer and transcription factors by binding to DNA responsive elements (RE, ERE, PRE, ARE).^103–105^ In addition, membrane bound G-protein-coupled receptors for all these sex hormones exist^106^ which modulate non-canonical TGFβ signalling pathways.

Oestrogens have strong implications in vascular diseases and promote cardiovascular protection.^107,108^ Frump *et al.*^109^ showed that 17β-oestradiol substantially improves right ventricular function in the Sugen-Hypoxia (SuHx) PH rat model, and they further linked ERα signalling in the right ventricle to protective adaptation in PAH in a BMPR2-dependent manner.^110^ Although less characterized than oestrogens, progestogens, and androgens are also cardiovascular active, and play a substantial role in vascular health and disease.^111–114^ While the effect of sex hormones on the (pulmonary) vasculature is well appreciated,^111,115,116^ the molecular mechanisms underlying their functions remain elusive. Both sex hormones and TGFβ family members exert a tight control of the vasculature also in pathogenic conditions like PAH.^26,116,117^ For comprehensive understanding of the TGFβ and sex-hormone crosstalk, we will summarize the molecular mechanisms described so far, mainly in vascular cells. Unfortunately, most mechanistic studies have been performed in non-vascular settings. Given that sex hormones act on many non-cardiovascular tissues, influencing systemic levels of circulating TGFβ components and hence indirectly the cardiovascular system, we will learn from studies performed in non-vascular tissues and discuss how the crosstalk between TGFβ signalling and sex hormones may be applicable to vascular biology and PAH.

### 4.1 Oestrogens

Oestrogen signalling involves several members of the TGFβ family pathway in a vascular context (*Table 1*). As such, transcriptome analysis of human umbilical vein endothelial cells (HUVECs) showed that the expression of *ACVRL1* (encoding ALK1), and latent-transforming growth factor beta-binding protein 3 (*LTBP3*) are increased in response to exogenous oestradiol, while *CAV2* (caveolin-2), a negative regulator of TGFβ1-induced ALK5/SMAD2/3 signalling in ECs,^132^ and *SMURF2* are decreased, partially overlapping the transcriptome of TGFβ1-stimulated cells.^119^ Additionally, administration of the selective oestrogen receptor modulator (SERM) Raloxifene increased the protein expression of ALK1 and endoglin in ECs,^118^ hence favouring SMAD1/5/8 over SMAD2/3 signalling. SERMs can have an agonistic and antagonistic effect, depending on the tissue type and availability of oestrogen receptors.^133^ These effects have been extensively studied in mammary and skeletal tissues but are underexplored in the cardiovascular system, which is evidently necessary in the context of PAH therapy.

**Table 1:** An overview of studies investigating transcriptional effects of the different sex hormones on targets within the TGFβ signalling cascade. The table shows increased or decreased expression, at which level it has been investigated, in which model or cell type and the specific metabolite used

Hormone	Expression ↑/↓	Level of expression	Model (tissue)/cell type	Metabolite	Ref.
Estrogens	↑ ALK1	mRNA and protein mRNA	HMEC-1 HUVECs	Raloxifene 17β-oestradiol	^118,119^
	↑ ALK5	Promoter Protein	Rat osteoblasts	Oestradiol	^120^
	↑ BMP2	mRNA	Mouse MSCs	17β-Oestradiol	^121^
	↑ BMP6	Promoter	Osteoblasts/MCF-7	17β-Oestradiol	^122^
	↑ BMPR2	Protein	RV Su-Hx rat RVCM WT/Su-Hx rats	17β-Oestradiol PPT	^110^
	↑ endoglin	mRNA and protein	HMEC-1	Raloxifene	^118^
	↑ LTBP3	mRNA	HUVECs	17β-Oestradiol	^119^
	↑ TGFβ3	Promoter and mRNA	Rat (bone)	17β-Oestradiol Raloxifene	^123^
	↓ BMPR2	mRNA Protein Protein	Wild-type mice HPASMC Su-Hx rat	17β-Oestradiol 17β-Oestradiol Anastrozole	^124–126^
	↓ ID	Protein	HPASMC	17β-Oestradiol	^125^
	↓ SMURF2	mRNA	HUVECs	17β-Oestradiol	^119^
Progestogens	↓ CTGF (TGFβ1 induced)	Promoter mRNA Protein	A549 (lung epithelial cells)	Progesterone	^127^
	↓ PAI-1 (TGFβ1 induced)	Promoter	MLECs (mink lung epithelial cells)	Progesterone	^127^
	↓ TAGLN (TGFβ1 induced)	Promoter mRNA Protein	A549	Progesterone	^127^
Androgens	↑ BMPR2	mRNA	PAH HPASMC	DHEA	^128^
	↑ BMP7	mRNA	Stellate cells	Testosterone	^129^
	↑ Chordin	mRNA (array)	Stellate cells	Testosterone	^129^
	↑ FST	Protein	Stellate cells	Testosterone	^129^
	↑ Noggin	mRNA (array)	Stellate cells	Testosterone	^129^
	↑ SMAD7	mRNA	Stellate cells	Testosterone	^129^
	↓ ACVR2A	mRNA	Stellate cells	Testosterone	^129^
	↓ BMP2	mRNA (array)	Stellate cells	Testosterone	^129^
	↓ BMP4	mRNA (array)	Stellate cells	Testosterone	^129^
	↓ Nodal	mRNA (array)	Stellate cells	Testosterone	^129^
	↓ PAI-1	mRNA (array)	Stellate cells	Testosterone	^129^
	↓ SMAD2/3	Protein	Rat (kidney)	Testosterone propionate	^130^
	↓ SMAD4	Protein	Rat (kidney)	Testosterone propionate	^130^
	↓ SMURF1	mRNA (array)	Stellate cells	Testosterone	^129^
	↓ TGFβ1	mRNA Protein	Stellate cells Rat (kidney)	Testosterone Testosterone propionate	^129,130^
	↓ TGFβR2	mRNA	Stellate cells	Testosterone	^129^
AMH	↓ ALK2	Protein	Lung epithelial cells	AMH (expressed)	^131^
	↓ ALK3	Protein	Lung epithelial cells	AMH (expressed)	^131^
	↓ BMPR2	Protein	Lung epithelial cells	AMH (expressed)	^131^

The plasma membrane G-protein-coupled oestrogen receptor (GPER, or GPR30) is an important mediator of oestrogen-induced signalling in vascular aetiologies.^134,135^ Interestingly, GPER activation by oestradiol or the GPER agonist G1 increased SMAD1/5/8 phosphorylation and the downstream target *ID1* in HUVECs.^136^ These effects can be inhibited by a G-protein pathway inhibitor, indicating a specific role for canonical GPER signalling. This study suggests for the first time a crosstalk between GPER and canonical TGFβ signalling in ECs, and therefore more research is encouraged. Activation of GPER induces Src, MAPK, and PI3K/Akt signalling via transactivation of the epidermal growth factor receptor (EGFR) pathway.^137^ GPER modulates hypoxia-inducible factor (HIF) and vascular endothelial growth factor (VEGF) signalling, which makes it an interesting receptor to target in the endothelium.^106^ In addition, oestrogen-GPER signalling enhances Notch-mediated epithelial-to-mesenchymal transition (EMT),^106,138^ a process resembling EndMT (functionally relevant in PAH, as described above). Importantly, all these non-canonical TGFβ signalling routes (*Figure 1*) have shown to impact PAH development.^139–142^

Oestrogens influence PAH disease development and are thought to be an important driver causing the sex bias in PAH. As such, decreased expression of an important 2-hydroxyestrogen (2-OHE) catalyst, CYP1B1, may be a second-hit favouring PAH development in female HPAH patients.^143^ In blood isolated lymphoblastoid cells, this enzyme showed a 10-fold decreased expression in affected compared to unaffected female *BMPR2* mutation carriers.^143^ As a follow-up, Austin *et al.*^144^ showed that female *BMPR2* mutation carriers have a 4-fold decreased disease penetrance when expressing the N453S CYP1B1 variant compared to wild-type. Further, they observed a decreased urinary 2-OHE/16α-OHE metabolite ratio in affected female *BMPR2* mutation carriers. Unexpectedly, the enzymatic function of CYP1B1 was unrelated to 2-OHE levels but predominantly caused by increased levels of 16α-OHE (although highly variable).^144^ This study therefore demonstrates the importance of oestrogen metabolites in PAH disease penetrance in women.

Indeed, Mair *et al.*^125^ found that basal BMPR2 protein levels in female non-PAH hPASMCs are lower compared to male cells. BMP4-induced pSMAD1/5/8 and *ID1/3* expression was lower in female than in male hPASMCs. Interestingly, administration of exogenous oestradiol to male hPASMCs decreased *ID1/3* expression to levels comparable to female cells.^125^ Consistently, oestrogen-ERα activation was reported to downregulate *BMPR2* expression in pulmonary microvascular ECs (MVECs) via an ERE in the promoter of *BMPR2*.^124^ Moreover, inhibition of oestrogen synthesis by the aromatase inhibitor anastrozole alleviated experimental PAH in a SuHx rat model by restoring BMPR2 expression.^126^ Conversely, in the right ventricle of multiple PH rat models and cultured rat right ventricle cardiomyocytes, E2-ERα signalling increased *BMPR2* expression.^110^ Further, basal BMPR2 levels were higher in female right ventricle samples compared to males. Interestingly, they showed a direct interaction between ERα and BMPR2, which improved cardiac function via Apelin upregulation. In this study, Frump *et al.* also showed a protective effect of E2, or an ERα agonist, by preventing PH disease development in multiple PH rat models, driven via this BMPR2/Apelin-axis. Compared to human control samples, IPAH patients showed decreased ERα levels in the right ventricle.^110^ Taken together, oestrogens decrease *BMPR2* expression in the vasculature but promote BMPR2 levels in the right heart. This cell type-dependent effect can explain female predominance and increased male severity in PAH.

**Figure 4:**
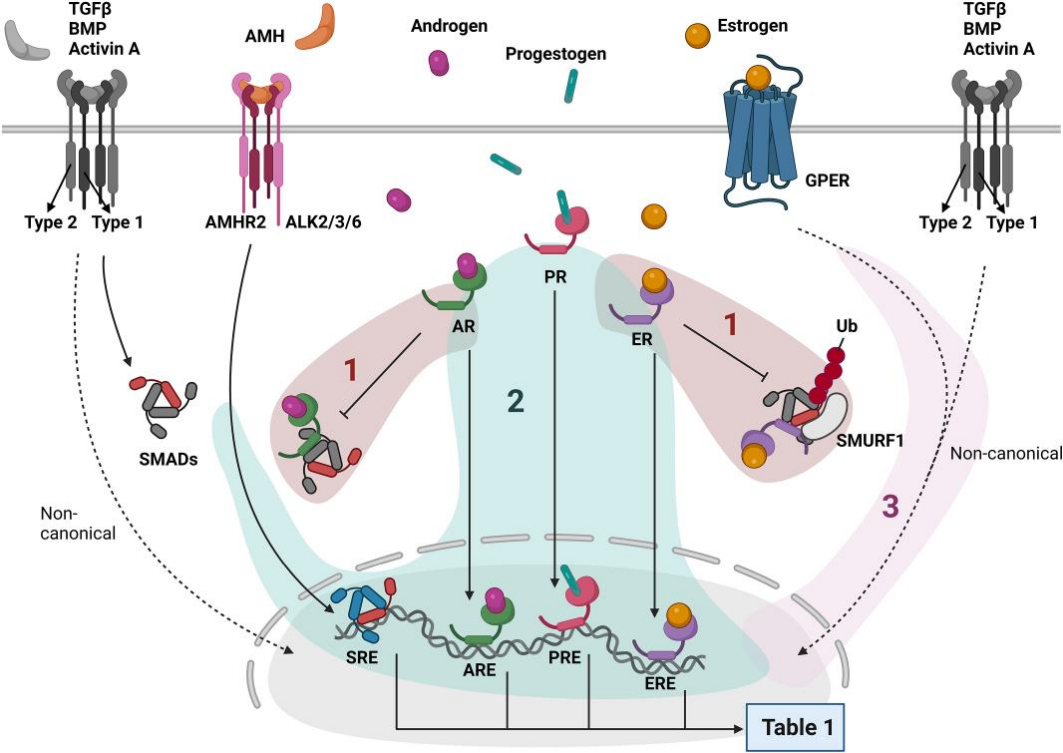
Signalling crosstalk of sex hormones and TGFβ signalling. The membrane permeable sex hormones androgens, progestogens, and oestrogens bind their nuclear receptors androgen receptor (AR), progestogen receptor (PR), and oestrogen receptor (ER), respectively. Oestrogens also bind the membrane receptor G-protein-coupled oestrogen receptor (GPER). Sex-hormones crosstalk on three different levels with TGFβ signalling. (1) The activated nuclear receptors can directly interact with SMADs to inhibit downstream signalling. Oestrogen-ER signalling has been associated with SMURF1-mediated proteasomal degradation of SMADs. (2) All sex-hormones have shown to regulate TGFβ target genes, via their corresponding responsive elements. (3) The oestrogen-GPER signalling cascade includes routes overlapping non-canonical TGFβ signalling routes. TGFβ, transforming growth factor-β; BMP, bone morphogenetic protein; AMH, anti-Müllerian hormone; AR/PR/ER, androgen/progestogen/oestrogen receptor; GPER, G-protein-coupled oestrogen receptor; SRE/ARE/PRE/ERE, SMAD/androgen/progestogen/oestrogen responsive element; SMAD, small mothers against decapentaplegic; SMURF, SMAD specific ubiquitin ligase.

Circulating sex hormones may be also secreted by and affect non-cardiovascular tissues, which in turn may impact the cardiovascular system indirectly. Through this angle, multiple studies have been performed using non-vascular cell models like MCF-7 and HEK293 that could help us to unveil the mechanistic crosstalk between TGFβ and sex hormones (summarized in *Table 1*). Researchers have shown that ERα/β can directly bind, inhibit, and recruit protein degradation systems (by e.g. SMURF1) to SMAD2/3 in an oestrogen-dependent manner (*Figure 4*).^145–148^ BMP stimulated SMAD1/5/8 phosphorylation was also reduced by oestrogen treatment in the same non-vascular cell lines.^149^ To add complexity to this oestrogen-TGFβ crosstalk, SMADs can also be a cofactor for sex-hormone receptor-mediated transcription.^150,151^ Evidently, as these studies made use of non-vascular cells, there is a need to confirm their findings towards vascular biology in the context of PAH.

In conclusion, accumulating evidence indicates that oestrogens can regulate canonical TGFβ signalling by directly altering the expression of TGFβ receptors and signalling modulators, at the transcriptional and protein level. Moreover, oestrogen signalling via GPER may indirectly modulate TGFβ non-canonical routes (*Figure 4*).

### 4.2 Progestogens

Progestogens may positively impact the cardiovascular system,^152^ by negatively regulating the hyperproliferation of ECs and SMCs.^112,153,154^ Progesterone induces a strong vasodilating response compared to oestradiol and testosterone in male and female rat coronary and pulmonary arteries *ex vivo.*^114^ Congruently, low progesterone levels correlate with increased risk of PAH in men.^155^ To date, a direct link between progestogens and TGFβ signalling (including BMPR2 regulation) in cardiovascular cells is underexplored. In epithelial cells, progesterone inhibits TGFβ1-induced SMAD3 phosphorylation in a dose-dependent manner,^127^ and antagonizes TGFβ1-mediated upregulation of the target genes *CTGF*, *transgelin*, and *PAI-1*. In human granulosa cells, BMP-15-induced signalling via BMPR2 and ALK6 was shown to suppress progesterone production,^156^ although likely indirectly. In addition, Activin A repressed progesterone synthesis in the reproductive system,^157,158^ which might explain low progestogen levels in male PAH patients,^155^ as Activin A plasma levels are increased.^89^ Similarly, BMP4 and BMP7 also suppressed progesterone synthesis in Granulosa-Lutein cells.^159^ The crosstalk between progesterone and TGFβ signalling is most likely cell type and context dependent.

In summary, although functional progesterone responses on vascular cells are well described, data regarding crosstalk between progestogens and TGFβ signalling in this context is lacking, and more research is needed to further understand the sex-related differences in PAH.

### 4.3 Androgens

Androgens have been proposed as a therapeutic treatment for PH,^116,160^ because of its quick beneficial vasodilatory effect on the pulmonary vasculature^21^ and its protective effect on right ventricle adaptation and function.^160,161^ Androgens classical mode of action involves gene transcriptional responses through intracellular binding to AR,^113,162,163^ expressed in PASMCs and ECs. The androgen-induced vasodilation response occurs within 20 minutes after androgen administration.^21,114^ As a direct effector, testosterone can antagonize calcium channels in SMCs, thereby triggering a fast cellular response, not mediated by classical AR-dependent gene transcription. The androgen metabolite DHEA is shown to restore cardiac remodelling and increase right ventricular function in rat models for experimentally induced PAH.^128,160^ Further, DHEA treatment of PAH patient-derived PASMCs increased *BMPR2* mRNA expression,^128^ explaining an increased disease penetrance in individuals with low DHEA-S levels.^164–166^ Therefore, DHEA (or DHEA-sulphate, -S) treatment is currently investigated in a clinical setting.^161^

Beyond the vasculature, androgens are described to modulate TGFβ signalling at multiple levels (*Figure 4* and *Table 1*). Also mechanistically, in prostate cancer cell lines such as LNCaP and PC3 cells, dihydrotestosterone (DHT)-induced AR transactivation can form a complex with SMAD3 and SMAD4, where SMAD3/AR complexes promote transcription via DNA binding to AREs, while SMAD3/SMAD4/AR complexes inhibit androgen target gene expression.^150^ Hayes *et al.*^167^ observed a repression of androgen target gene expression by SMAD3/AR complexes, by direct binding of the MH2 domain of SMAD3 with the transcription activation domain of the AR. Interestingly, the androgen-driven inhibitory effects on gene transcription are not specific for the TGFβ branch of the family, but also BMP signalling and its downstream targets are inhibited upon DHT treatment in e.g. intestinal stromal cells.^168^ Furthermore, phosphorylated SMAD1 interacts with AR to suppress its transcriptional function,^169^ indicating that androgens may regulate both TGFβ and BMP signalling pathways and vice versa (*Figure 4*).

In conclusion, androgens and TGFβ crosstalk via direct AR and SMAD interactions and indirectly via transcriptional regulation through AREs (*Figure 4*). The vast majority of these data result from studies using prostate cancer or other non-vascular models but may very well be applicable to PAH. For example, testosterone administration increased the expression of the circulating TGFβ regulators Follistatin, Chordin, and Noggin expression in muscle stellate cells^129^ (*Table 1*), which may impact distant organs, including the heart and the pulmonary vasculature. PAH patients exhibit increased Activin A and Follistatin circulating levels,^89^ and Activin A levels correlate with increased mortality. Higher androgen-mediated Follistatin in males could potentially suppress high amounts of Activin A in PAH and might contribute to the lower prevalence in men.^170^ The decrease in androgens with age would lead to decreased Follistatin levels with increased active Activin A levels and disturbed TGFβ and BMP signalling balance as consequence. In line, the sex-biased disease prevalence in PAH also decreases upon ageing.^12^ Following this hypothesis, one might warrant the prescription of (Activin A) ligand traps like Sotatercept. Indeed, as described earlier, clinical trials have been performed treating Sotatercept to PAH patients with striking results.^8,171^

Taking into consideration the TGFβ/BMP balance and the effects sex hormones have on TGFβ signalling components, including BMPR2, one could assume that BMPR2 expression levels are higher in men compared to women. Low androgen levels with a corresponding drop in *BMPR2* expression could initiate PAH development, as low DHEA-S levels are correlated with worse disease outcome in male PAH patients.^166^ Further, high androgen-driven Follistatin levels in men might protect from pathogenic signalling by e.g. Activin A in PAH. Taken together, this delineates a higher incidence in PAH development in predominantly younger women but also a more severe disease outcome in men with low DHEA levels.^166^

### 4.4 Anti-Müllerian hormone

AMH is expressed in follicular sertoli and ovarian granulosa cells and is known to be a circulating hormone throughout life, although declining with age. AMH is a TGFβ family member that binds its dedicated TGFβ Type II receptor AMHR2,^172^ also expressed in the human heart.^173^ Associated Type I receptors include ALK2, -3 and -6, thereby involving BMP-like downstream signalling (*Figure 1*).^37,172^ Although typically linked with sexual dimorphisms^174^ and female fertility, other studies indicate AMH to have cardiovascular regulatory properties. Since 2012, high levels of AMH have been correlated with cardiovascular protection,^175^ decreased plaque diameter in non-human primates,^176^ and decreased male aortic diameter, which are all risk factors for aneurysm.^177^ More recently, in the Doetinchem Cohort Study, they found that decreasing AMH trajectories are associated with a substantial elevated risk of CVD in women.^178^

A potential role of AMH in PAH was recently suggested in a case report study^179^ describing a novel loss-of-function *BMPR2* mutation in exon 2 associated with IPAH development. The resulting BMPR2 mutant protein is unable to translocate to the plasma membrane. Comprehensive analysis of the TGFβ/BMP signalling signature in peripheral blood mononuclear cells (BPMCs) of this patient confirmed low BMPR2 expression levels, and increased expression of AMHR2, ALK1, ALK3, and ALK6 protein levels, whereas TGFβ receptors remained unchanged.^179^ Noteworthy, increased SMAD1/5 and SMAD2/3 phosphorylation was observed upon BMP2 and TGFβ stimulation. Furthermore, mRNA expression of the BMP target genes *ID1*, *SMAD6*, and *STAT1* was increased, suggesting that BMP signalling was not compromised due to the *BMPR2* mutation, at least in PBMCs. The expression of AMHR2 in PBMCs supports the hypothesis that AMH affects inflammation responses and therefore influences PAH. Indeed, higher circulating AMH levels has been correlated with the reduced inflammation marker C-reactive protein in men.^180^ Disturbed inflammatory responses have been proposed as an additional driver of PAH development,^181^ therefore, reducing inflammation via increased AMH signalling in *BMPR2* mutant carriers might be beneficial in PAH. In this case report however, increased AMHR2 not necessarily proves increased signalling as functional AMHR2 ligands activity was not quantified.

Studies using lung cancer epithelial cells reported a crosstalk between AMHR2 and BMPR2 causing enhanced SMAD2/3 phosphorylation upon loss of AMH or AMHR2,^131^ possibly via mixed-heteromeric receptor complexes driven by BMP ligands.^93^ Correspondingly, in these cancerous epithelial cells, siRNA depletion of *AMH* or *AMHR2* drives EMT,^131^ suggesting inhibitory functions of AMH in EMT. Early in life, males show higher AMH levels than females, but women have higher AMH levels throughout life.^177^ To date, relevant data in relation to the pulmonary vasculature are lacking, but if the mechanisms described above for AMH are applicable to vascular cells too, unravelling the role of AMH in the vasculature might help understand PAH disease development.

### 4.5 Sex hormonal therapy and the clinic

The crosstalk between oestrogens and androgens and the TGFβ signalling family is relatively well described in the vascular system. The findings described in previous chapters indicated a protective effect of androgens, by increasing *BMPR2* expression and circulating Follistatin levels, and oestrogens being an additional risk factor, by decreasing BMPR2 levels in the vasculature but cardioprotective in the heart. Correspondingly, targeting sex-hormone signalling in PAH is a strategy applied within the clinic by multiple groups.

Baird *et al.* showed that lower levels of dehydroepiandrosterone-sulphate (DHEA-S, a prohormone for androgens and oestrogens) and higher levels of E2 were associated with severe PAH in men^164^ and in post-menopausal women.^165^ This profile caused a worsened disease outcome, suggesting substantial roles of these sex hormones in disease progression and response.^164^ In a recent study analysing a large Dutch PAH cohort, low DHEA-S levels in male and female PAH patients were confirmed.^166^ These studies validated a clinical trial to evaluate the effect of DHEA-S administration in PAH (EDIPHY: NCT03648385).^161^ Targeting high oestrogen levels also seems a possible treatment option for PAH, as oestrogen inhibition by anastrozole (aromatase inhibitor) and fulvestrant (ER antagonist) prevented and reversed PAH development in BMPR2 mutant mice.^182^ A small proof-of-concept trial using fulvestrant on five PAH patients showed an increasing trend of the primary outcome 6-minute walking distance comparing baseline with 9 weeks of treatment, although not significant (NCT02911844).^183^ Two clinical studies are being conducted using anastrozole in PAH. The first small Phase 2 clinical trial of anastrozole in PAH patients showed a 40% reduction of oestrogen plasma levels, a good safety profile and a significant increased 6-minute walking distance. However, other PAH clinical outcome measures remained unchanged (NCT01545336).^184^ A larger follow-up trial has been recently performed (PHANTOM: NCT03229499). While we still wait for the final data to be published, the preliminary results presented at the American Thoracic Society International Conference 2023 revealed no significant improvement in 6-minute walking distance after 6 months, NT-proBNP levels or echocardiographic parameters in individuals treated with anostrozole.^185^ Importantly, oestrogens show a protective effect on the right heart by increasing BMPR2 levels.^110^ Therefore, this might raise concerns when applying anti-oestrogen therapies. However, PHANTOM showed that decreasing oestrogen levels did not have adverse effects on the right heart of PAH patients. Of course, potential systemic effects of anti-oestrogen therapy should be carefully evaluated, particularly when treating reproductive aged women.

In this regard, pregnancy has been associated with increased risk of PAH development in *BMPR2* mutation carriers, as patients have been diagnosed with PAH after pregnancy.^186^ Disease severity is also higher peri- and post-partum,^187^ resulting in a mortality of pregnant PAH patients of around 11–25%.^2^ These observations can easily be linked to drastic haemodynamic changes during pregnancy,^187^ but the long-term effects of hormonal changes are often not considered. As such, oestrogens and progestogens rise dramatically during pregnancy. As already described, this affects the TGFβ family signalling pathway in different manners. Hence, sex-hormonal changes during pregnancy might enhance TGFβ signalling dysregulation (by an additional drop of BMPR2 levels in the vasculature) and subsequent PAH development and severity.

Taken together, these studies underline the importance of sex hormones in PAH disease initiation and progression (in pregnancy) and set the stage for clinical (anti-)hormone therapies for PAH, although context-dependent cellular and molecular mechanisms driving these effects are still incompletely understood.

## 5. Genetic-related sex differences and the TGFβ signalling family

The X and Y sex chromosomes contain specific genetic information which might differentially regulate the TGFβ signalling family in males and females. Although most of the genes expressed from the Y-chromosome encode for proteins required during gonad development, some factors also have roles outside the reproductive system. In females, expression levels of genes located on the X-chromosome are regulated by the inactivation of one of the two X-chromosomes. As we will discuss below, in some occasions this process can be disturbed, leading to enhanced gene expression due to increased genetic load. In this section, we elaborate on X- and Y-linked genes in relation to the TGFβ signalling family in PAH.

### 5.1 Y-chromosomal expression

The Y-chromosome is a relatively small chromosome containing a low number of genes in comparison with other mammalian chromosomes. There are 568 genes harboured on the Y-chromosome, of which only 71 have protein encoding potential.^188^ Multiple genes encode proteins of the same protein families, leaving only 27 non-related proteins encoded on the Y-chromosome. In a mouse model for PAH, Umar *et al.*^25^ found that the Y-chromosome protects disease development, unrelated to gonadal sex (testes or ovaries), suggesting an important role for Y-chromosomal expression in preventing PAH development. Of all Y-chromosomal genes, the sex-determining region Y (*SRY*) gene is the most studied.^189^ SRY is a DNA-binding transcription factor regulating gene expression at the early initiation of testes development, but SRY also functions outside the reproductive system.^190^ As such, SRY directly binds the promoter of *BMPR2* to upregulate *BMPR2* expression in PAH fibroblasts.^191^ As females lack SRY, this *BMPR2* transcriptional regulation does not occur. Correspondingly, *BMPR2* mRNA levels in male PAH patient-derived lymphocytes are higher compared to female equals.^124^ Further, SRY may indirectly modulate the TGFβ family signalling by interacting with AR thereby dampening testosterone-induced transcription.^192^

**Figure 5:**
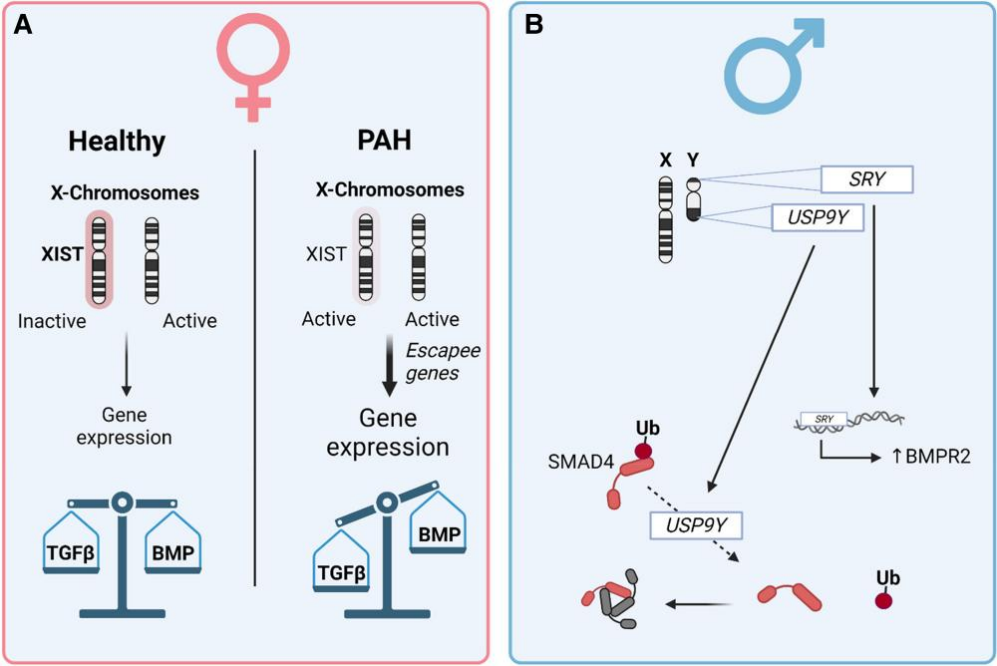
Genetics sex-related differences on the TGFβ signalling family in health and PAH. (*A*) In females, proper X-chromosome inactivation results in healthy genetic output leading to a balanced TGFβ/BMP signalling. However, disturbances in X-chromosome inactivation results in dysregulated genes (*escapees*) and increased genetic output which might cause a diseased disbalance in TGFβ/BMP signalling. (*B*) In males, SRY has been linked to increased BMPR2 expression, while USP9Y is an ubiquitin-dependent hydrolase that targets SMAD4. TGFβ, transforming growth factor-β, BMP, bone morphogenetic protein; SMAD, small mothers against decapentaplegic; SRY, sex-determining region of Y; USP9Y, ubiquitin specific peptidase 9 Y-linked; BMPR2, BMP receptor Type 2.

Of all the genes found on the Y-chromosome in PAH patients, eight genes showed decreased expression in diseased lung tissues.^25^ One of these genes is *USP9Y*, a ubiquitin-associated hydrolase preventing ubiquitin-dependent degradation of proteins including SMAD4, thereby increasing TGFβ signalling (see reference ^193^ and ENSG00000114374). Another downregulated Y-linked gene in PAH lungs is the ATP-dependent RNA helicase *DDX3Y.*^25^ Although DDX3Y interacts with SMAD2 and SMAD3,^194^ the functional consequence of this interaction is unknown. In summary, Y-specific expression profiles may alter the signal transduction induced by TGFβ family members (*Figure 5B*) and might prevent the initiation and progression of PAH. How these interactions with the TGFβ family results in changes of cellular behaviour needs still to be deciphered.

### 5.2 X-chromosome inactivation

The X-chromosome contains over 1200 genes. In females, the expression of X-linked genes is tightly regulated by X-chromosomal inactivation. This process is necessary for genetic dosage, leading to similar gene expression levels of X-linked genes in female XX cells compared to XY male cells.^195^ Silencing of the X-chromosome is mediated by the long non-coding RNA (lncRNA) antisense pair X-inactive specific transcript (XIST) and TSIX (XIST, opposite strand). While *XIST* shields (thereby silences) one of the X-chromosomes, *TSIX* impairs the inactivation of the active X-chromosome through complementary binding to XIST. Furthermore, epigenetic modifications of the *XIST* locus can cause XIST silencing.^196^ In addition, the lncRNA X-active specific transcript (XACT) coats the active X-chromosome and also antagonizes *XIST.*^197^ Most genes on the inactivated X-chromosome remain silenced; however, 15–25% of X-linked genes escape this silencing process (known as ‘escapees’).^198^ These escapees have been linked to sex differences in diseases like auto-immune diseases and cancers.^199^

Recently, in the EH_itsn_-KO^ITSN+/−^ PAH mouse model for plexiform arteriopathy, Xist expression levels were increased in female PAH mice compared to the male mice or female WT mice.^200^ Noteworthy, female EH_itsn_-KO^ITSN+/−^ mice showed worsened vascular remodelling compared to their male equals. While no difference in Xist levels were observed in the SuHx PAH rat model, increased Xist expression was observed in human female PAH lungs compared to healthy subjects. Taken together, the upregulations of the lncRNA Xist/XIST may explain the sexual dimorphism in vascular remodelling and therefore highlights the importance of X-chromosome inactivation in the sex bias in PAH.

Several studies suggest an interplay with Xist and BMP/TGFβ signalling. Genetic knockdown of *ACVR1B* (ALK4), *BMPR2*, and *SMAD2* inhibits the expression of *Xist* in mouse fibroblasts.^201^ BMP signalling was found to induce and maintain the expression of XIST, while TGFβ signalling served as an antagonist. Furthermore, TGFβ signalling induced TSIX expression in dermal fibroblasts.^202^ Although specific XIST/TSIX expression levels are suggestive for X-chromosomal silencing, deeper comprehensive studies are needed for conclusive results. Nevertheless, dysregulation of TGFβ/BMP signalling could impact the chance of genes on the X-chromosome to escape gene silencing, thereby contributing to sex differences in PAH pathology.

The genetic impact on PAH development suggest a protective role for specific genes expressed from the Y-chromosome.^25^ The Y-chromosomal expressed SRY transcription factor upregulates *BMPR2* expression in PAH fibroblasts.^191^ As discussed above, TGFβ signalling can influence X-chromosomal inactivation in females, further enhancing TGFβ signalling disbalance in PAH. These observations strengthen the link between sex hormones, sex-related genetics, disturbed TGFβ signalling, and PAH disease development.

## 6. Hereditary haemorrhagic telangiectasia

The genetic background and disease aetiology in Hereditary Hemorrhagic Telangiectasia (HHT) (or Rendu–Osler–Weber syndrome) and HPAH patients sometimes overlap.^203^ Interestingly, there is also a sex bias observed in HHT although this is less pronounced compared to PAH. Therefore, many findings in this review are also relevant in a HHT context, which we shortly highlight in this section.

HHT is a vascular disorder presenting with malformed vessels leading to telangiectasia (*spider veins*), haemorrhages, and arteriovenous malformations (AVMs).^204^ Similarly as HPAH, HHT originates in people harbouring loss-of-function mutations in genes encoding BMP receptors, i.e. *ACVRL1* (ALK1: HHT2) and *ENG* (endoglin: HHT1).^98,205^ It is thought that decreased BMP signalling causes endothelial dysfunction, leading to the malformed vasculature in HHT.^206,207^ Sex differences in HHT present mainly by more severe symptoms in women compared to men (increased pulmonary and hepatic AVMs),^208,209^ although some small registry studies describe a female predominance.^210–212^

In this review, we explored sex differences in the TGFβ signalling family in PAH, but our discussion may have implications for HHT too. For instance, administration of Raloxifene increases ALK1 and ENG expression in ECs^118^ and is therefore proposed as treatment option for HHT (reviewed in reference ^213^). Another SERM, Tamoxifen, showed promising effects in a clinical trial reducing severe epistaxis.^214^ There is a marked influence of sex in pulmonary and hepatic vascular malformations in HHT, suggesting organ or tissue-specific features in comparison with other organs.^215^ It might be that expression levels of sex-hormone receptors in hepatic or pulmonary ECs makes these cells more sensitive to circulating sex hormones. This review highlights three levels on which sex hormones can alter TGFβ signalling (*Figure 4*). Further research on these organ-specific endothelial effects is warranted to delineate the sex bias in HHT.

## 7. Discussion and concluding remarks

PAH is a cardiovascular disease with a clear sex bias towards increased female predominance and more severe male phenotype. The molecular causes of this bias are incompletely understood. This review therefore explored sex differences in the TGFβ signalling family to understand the sex bias in PAH (and by extension in HHT).

We have emphasized that hormonal and genetic sex differences may regulate the TGFβ signalling family in different ways to contribute to PAH. Noteworthy, many of the mechanistic findings described above originate from non-vascular cell models, hence translation into PAH should be done carefully. Future studies should be performed aiming to investigate sex-specific effects on the TGFβ signalling family in a cardiovascular setting. Often, sex-related genetics are not taken into account while investigating sex hormonal effects on TGFβ signalling. For instance, researchers should include karyotypes of the cells or tissues studied. We further stress the importance of implementing sex-related genetics in sex-hormone-based studies.

In the meantime, we can anticipate that personalized treatments will progressively become more relevant in clinical decision-making, and therefore sex-related components need to be addressed accordingly. We highlight sex-specific features like hormones and genetic differences in relation to the TGFβ signalling pathway in pulmonary vascular diseases. These findings could implicate differential treatments based on sex, e.g. hormonal therapy like tamoxifen, raloxifene, anastrozole, or DHEA-S, of which the latter two clinical trials are discussed in this review (Section 4.5). These trials are eligible for all sexes although, depending on the study outcomes, sex-customized treatments should not be overlooked. Adverse effects of hormone therapies might be overcome by the development of next-generation SERMs like LY2066948.^133,216^ Unfortunately, anastrozole (anti-oestrogen) therapy in PAH showed lack of efficacy following the preliminary clinical data.^185^ Conversely, pre-clinical evidence shows that oestrogen administration also ameliorates PAH outcome in a tissue-specific manner, by targeting the right heart.^110^ Oestrogen therapy targeting the heart, as an organ-specific treatment, might therefore be a promising treatment option, especially in men showing less right ventricular adaptation.

Overall, sex-specific differences in the TGFβ signalling family potentially explain sex differences in PAH. Many aspects of sex-related crosstalk with the TGFβ signalling family within the cardiovascular system are incompletely understood and more research is therefore warranted. Sex-specific determinants are becoming increasingly important for biomarker identification, drug development and therefore, to find a definitive cure for PAH.
